# Clinical practice guidelines for the foot and ankle in rheumatoid arthritis: a critical appraisal

**DOI:** 10.1186/s13047-016-0167-0

**Published:** 2016-08-19

**Authors:** Kym Hennessy, James Woodburn, Martijn Steultjens

**Affiliations:** 1 Institute for Applied Health Research, School of Health & Life Sciences, Glasgow Caledonian University, Glasgow, UK; 2 Department of Podiatric Medicine, School of Science & Health, Western Sydney University, Sydney, Australia

**Keywords:** Rheumatoid arthritis, Clinical practice guidelines, Foot, Ankle

## Abstract

**Background:**

Clinical practice guidelines are recommendations systematically developed to assist clinical decision-making and inform healthcare. In current rheumatoid arthritis (RA) guidelines, management of the foot and ankle is under-represented and the quality of recommendation is uncertain. This study aimed to identify and critically appraise clinical practice guidelines for foot and ankle management in RA.

**Methods:**

Guidelines were identified electronically and through hand searching. Search terms ‘rheumatoid arthritis’, ‘clinical practice guidelines’ and related synonyms were used. Critical appraisal and quality rating were conducted using the Appraisal of Guidelines for Research and Evaluation (AGREE) II instrument.

**Results:**

Twenty-four guidelines were included. Five guidelines were high quality and recommended for use. Five high quality and seven low quality guidelines were recommended for use with modifications. Seven guidelines were low quality and not recommended for use. Five early and twelve established RA guidelines were recommended for use. Only two guidelines were foot and ankle specific. Five recommendation domains were identified in both early and established RA guidelines. These were multidisciplinary team care, foot healthcare access, foot health assessment/review, orthoses/insoles/splints, and therapeutic footwear. Established RA guidelines also had an ‘other foot care treatments’ domain.

**Conclusions:**

Foot and ankle management for RA features in many clinical practice guidelines recommended for use. Unfortunately, supporting evidence in the guidelines is low quality. Agreement levels are predominantly ‘expert opinion’ or ‘good clinical practice’. More research investigating foot and ankle management for RA is needed prior to inclusion in clinical practice guidelines.

## Background

Clinical practice guidelines are systematically developed recommendations that are used to inform stakeholders about appropriate healthcare and assist in decision making for specific clinical situations [1]. Clinical practice guidelines provide the basis for evidence-based best practice in various clinical situations [2]. Furthermore, guidelines can also help to inform changes to healthcare policy, as policy makers look for healthcare to be more efficient and consistent [3]. Whilst having access to clinical practice guidelines has many benefits, the overall benefit is only achievable if the guidelines are good quality [2, 4]. Therefore, appropriate robust methodologies for developing and appraising guidelines are needed [1].

There is a high prevalence of foot involvement in rheumatoid arthritis (RA) with over 90 % of patients reporting foot pain during the course of the disease [5, 6]. Over 60 % of patients report walking disability and foot involvement impacts negatively on health-related quality of life [7, 8]. There are many clinical practice guidelines currently available specifically related to the management of RA. The majority are concerned with pharmacological management of RA. Although, some guidelines take a more multidisciplinary approach to management, and include foot and ankle care. However, management of the foot and ankle in RA is still under-represented in the overall management of RA. This is unfortunate as foot and ankle care continues to play a large role in the holistic management of RA as active disease and associated symptoms can persist even after reaching clinical remission [9–11]. Whilst, some foot and ankle care guidelines are currently available, the quality of these guidelines has never to our knowledge been appraised.

Therefore, the aim of this review was to identify and critically appraise clinical practice guidelines that included management of the foot and ankle in RA to inform current practice.

## Methods

### Search strategy

Guidelines were identified electronically in the following databases: Medline (1950 to August 2015), Embase (1979 to August 2015), CINAHL (1981 to August 2015), AMED (1987 to August 2015), PEDro (1990 to August 2015), and the Cochrane Library (1974 to August 2015). Guidelines were also identified by hand searching the reference lists of the electronically identified studies and the researchers’ own literature databases.

A two-way search strategy was employed using ‘rheumatoid arthritis’ with ‘clinical practice guidelines’ and related synonyms. Search terms were determined primarily by the researcher in consultation with academic supervisors. The search strategy specifically did not include foot or ankle, and related terms or specific treatment terms such as podiatry. This was due to many clinical practice guidelines including foot and ankle care without this being specifically highlighted in the title or keywords. Thus, a number of applicable guidelines could have been missed by the electronic search if the search strategy was more specific. The search strategy was a combination of Medical Subject Heading (MeSH) terms and text-words. MeSH terms were used when available and text-words were used when MeSH terms were unavailable or when a specific database did not facilitate the use of MeSH terms. Associated wildcards and truncations for each database were also used. The search strategy was formulated in Medline and was adapted to make it applicable to the other databases.

The **Search Strategy in Medline (EBSCO)** (adapted for other electronic databases):

1. (MH “Arthritis, Rheumatoid” **OR** [“Rheumatoid” **AND** “Arthritis”])
2. (MH “Guideline” **OR** MH “Practice Guidelines” **OR** “Guidelines” **OR** “Clinical Practice” **OR** “Practice” **OR** “Management” **OR** “Treatment” **OR** “Intervention” **OR** MH “Therapy”)
3. **1 AND 2**

MH = MeSH Heading

### Study selection criteria

Clinical practice guidelines that included foot and ankle care were selected. This included specific foot and ankle guidelines and general management guidelines that included the foot and ankle. No restriction was placed on language. No restrictions were imposed on year of publication. However, only the most recent version of a specific guideline was included. Any superseded version was excluded. Additionally, clinical practice guidelines that involved other rheumatological conditions were included provided there was specific RA related guideline content. All foot and ankle clinical practice guidelines were selected for further analysis. No limitations were imposed on who provided the management. Diagnostic assessment guidelines were also included as assessment is a vital part of overall management.

The abstracts of all studies found electronically and through hand searching were compared to the inclusion criteria. The electronic abstracts were exported from the databases and collated into a single document in Microsoft Office Word 2007 (Microsoft Corporation, Redmond, Washington, USA). This document also had the abstracts identified by hand searching added to it. The selection of abstracts (from the aforementioned collated document) that appeared to meet the inclusion criteria was conducted by two reviewers (KH and MPMS) independently. For the abstracts identified by either reviewer (or both reviewers) that appeared to meet the inclusion criteria, full-text guidelines were obtained and compared to the inclusion criteria independently prior to quality assessment. Only full-text guidelines were included for quality assessment. Additionally, due to the pragmatic approach of the search, clinical practice guidelines that were published as books rather than journal articles were also included.

### Quality assessment

Guideline quality was assessed using the Appraisal of Guidelines for Research and Evaluation (AGREE) II instrument. This is an appraisal instrument that assesses the methodological rigour and transparency used when a guideline is developed; components of the overall recommendations; and factors that influence adherence to the guidelines [12]. This appraisal instrument is the latest version of the original AGREE instrument, which is a valid and reliable tool for guideline appraisal [13]. It was originally developed, by an international collaboration, through a multi-stage procedure that included item generation, selection and scaling, field testing and refinement procedures [14]. It is also generally accepted as the standard for guideline appraisal [15]. The newest iteration of the AGREE instrument, the AGREE II instrument, was developed in response to issues that arose from the original instrument, such as the need for refinement of the purpose, response scale and instrument items [13]. Similarly to the original AGREE instrument, the AGREE II instrument was developed by an international collaboration through a multi-stage procedure [13, 16]. Whilst construct validity has been tested and established [17], the AGREE II instrument has yet to have been tested for reliability. However, it was decided that the AGREE II instrument was the appropriate appraisal instrument tool due to being the newest version of the instrument, increased construct validity compare to the original instrument [17], and the changes made were done to increase the measurement properties of the instrument [13, 18]. The AGREE II appraisal instrument is a 23-item tool where items are divided between six different domains. The six domains are: scope and purpose; stakeholder involvement; rigour of development; clarity of presentation; applicability; and editorial independence [12]. Quality assessment was performed by two reviewers (KH and MPMS) independently. Any disagreement was resolved by a third independent reviewer (JW).

### Data extraction and evidence grading

**Fig. 1:**
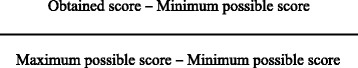
Domain score calculation for the AGREE II Instrument

The AGREE II instrument, as part of the overall appraisal process, includes a grading system. A seven point Likert scale of ‘strongly disagree’ to ‘strongly agree’ is used to grade each individual item of the AGREE II instrument. Strongly disagree is awarded when no information or poorly reported information is present for a specific item. Strongly agree is awarded when the quality of reporting is exceptional and the full criteria and considerations are met. Each domain is scored by combining all reviewers’ scores for each item and scaling the total score as a percentage of the maximum possible score (Fig. 1).

Whilst a quantitative grading can be given to each domain and can help to inform whether a guideline should be recommended for use, no specific criteria for high and low quality ranking is provided [12]. For this critical appraisal, overall quality of the guideline was determined in the same way that each domain was quantified, in that each domain score was combined and the overall percentage of the maximum was determined. The determination of whether a guideline was high or low quality was informed by the overall quality score and made at the discretion of the reviewers (following discussion). High quality guidelines were classified as those that would be recommended without modifications. Low quality guidelines were classified as those that would not be recommended. An intermediate category of recommended with modifications was used when it was decided (through reviewer discussion) that a guideline should be recommended for use, provided issues with the guideline development identified during appraisal were rectified. Those guidelines that fell within the intermediate category of recommended with modifications were classified as high or low quality at the discretion of the reviewers, and this was based on the amount and type of modifications required.

## Results

**Fig. 2:**
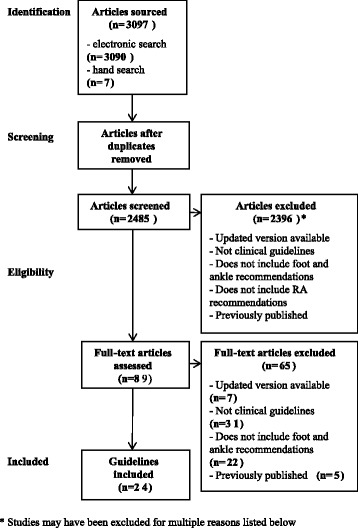
PRISMA literature search flowchart diagram

A total of 3097 clinical practice guidelines were retrieved using the detailed search strategy. Figure 2 outlines the flow chart used to identify clinical practice guidelines for inclusion (adapted from [19]). The inclusion criteria were met by 24 guidelines. This was from a total of 46 general clinical practice guidelines identified. Ten guidelines were high quality with five of these guidelines rated high quality, even though modifications need to be made to them. Of the high quality guidelines that were recommended with modifications, there was a maximum of two domains identified that required changing for each guideline. These identified domains were applicability (as guidance on implementation and cost was omitted), editorial independence (as competing interests and funding was omitted), and/or scope and purpose (greater clarity required). Four of the guidelines were for early RA and six of the guidelines were for established RA. Fourteen guidelines were low quality. However, seven of these guidelines would still be recommended if modifications were made. There was a maximum of three domains identified that required changing for each low quality guideline that was recommended with modifications. These identified domains were rigour of development (greater clarification of the process to develop the guidelines required), scope and purpose (more depth about the reason for the guideline needed), clarity of presentation, stakeholder involvement (as not all stakeholders were involved in the guideline development), applicability (as guidance on implementation and cost was omitted), and/or editorial independence (as competing interests and funding were omitted). Of the recommended low quality guidelines, one was for early RA and six were for established RA. Two guidelines were specifically for foot and ankle management alone. Within the included general guidelines, foot and ankle care recommendations only accounted for small sections of the guidelines, ranging from one sentence to one page. The individual domain and overall assessments, usage recommendations, and quality levels are shown in Table 1. Five recommendation domains were identified for early RA. They were multidisciplinary team care, access to foot healthcare, foot health assessment/review, orthoses/insoles/splints, and therapeutic footwear. Six recommendation domains were identified for established RA. They were multidisciplinary team care, access to foot healthcare, foot health assessment/review, orthoses/insoles/splints, therapeutic footwear, and other foot care treatments. The foot care related recommendations from each guideline recommended for use and the grade of each these recommendation (based on level of evidence) are described in Table 2 for early RA and Table 3 for established RA.

**Table 1:** Quality assessment of included guidelines

Author, Year	Scope & Purpose	Stakeholder Involvement	Rigour of Development	Clarity of Presentation	Applicability	Editorial Independence	Overall Assessment	Recommend (Y/M/N)	Quality Level
ACR 2002 [23]	42 %	42 %	34 %	44 %	35 %	58 %	**42 %**	N	Low
ARMA 2004 [33]	56 %	78 %	**42 %**	64 %	**44 %**	33 %	**58 %**	M	Low
Brosseau et al. 2004 [40]	56 %	58 %	63 %	67 %	**10 %**	38 %	**58 %**	M	High
Colebatch et al.2013 [41]	**31 %**	**33 %**	61 %	75 %	**9 %**	58 %	**50 %**	M	Low
Combe et al. 2007 [20]	67 %	44 %	56 %	61 %	8 %	13 %	**42 %**	N	Low
da Mota et al. 2012 [24]	53 %	33 %	28 %	75 %	15 %	46 %	**33 %**	N	Low
DSR 2009 [42]	69 %	78 %	67 %	75 %	56 %	63 %	**75 %**	Y	High
Forestier et al. 2009 [43]	56 %	47 %	57 %	78 %	**2 %**	46 %	**42 %**	M	Low
Gossec et al. 2005 [44]	64 %	58 %	56 %	67 %	**10 %**	**17 %**	**42 %**	M	Low
Gossec et al. 2006 [45]	61 %	**36 %**	61 %	75 %	**0 %**	**21 %**	**50 %**	M	Low
Hodkinson et al. 2013 [21]	61 %	64 %	26 %	67 %	27 %	17 %	**42 %**	N	Low
Kennedy et al. 2005 [46]	67 %	42 %	**44 %**	**69 %**	44 %	75 %	**50 %**	M	Low
Luqmani et al. 2006 [47]	89 %	64 %	55 %	75 %	50 %	67 %	**67 %**	Y	High
Luqmani et al. 2009 [48]	92 %	64 %	61 %	81 %	56 %	54 %	**67 %**	Y	High
NICE 2009 [49]	92 %	86 %	74 %	78 %	65 %	58 %	**75 %**	Y	High
Physicians of India 2002 [25]	56 %	31 %	35 %	58 %	8 %	17 %	**25 %**	N	Low
PRCA 2008 [34]	61 %	75 %	42 %	69 %	**38 %**	**33 %**	**58 %**	M	High
RACGP 2009 [50]	86 %	61 %	75 %	75 %	**38 %**	**42 %**	**58 %**	M	High
Rheum Found Japan 2004 [26]	44 %	36 %	34 %	42 %	13 %	17 %	**25 %**	N	Low
Schneider et al. 2011 [51]	67 %	58 %	68 %	69 %	29 %	71 %	**67 %**	Y	High
SER 2011 [52]	**50 %**	39 %	84 %	81 %	**25 %**	67 %	**67 %**	M	High
SIGN 2011 [53]	81 %	72 %	81 %	67 %	50 %	**25 %**	**67 %**	M	High
Walsh et al. 2007 [22]	44 %	56 %	24 %	67 %	29 %	33 %	**42 %**	N	Low
Williams et al. 2011 [35]	67 %	56 %	**36 %**	61 %	**31 %**	38 %	**42 %**	M	Low

Bold % in individual domains are domains requiring modification; Y = Yes; M = Yes with modifications; N = No

**Table 2:** Recommended early RA guidelines

Guideline	Multidisciplinary team care	AAccess to foot healthcare	Foot health assessment/review	Orthoses/insoles/splints	Therapeutic footwear
Clinical practice guidelines for the use of non-pharmacological treatments in early rheumatoid arthritis [45]			Metatarsal pain and/or foot alignment abnormalities should be looked for regularly (GCP)	Appropriate insoles should be prescribed if needed (GCP)	
BSR and BHPR Guidelines for the management of rheumatoid arthritis (the first 2 years) [47]	Podiatry is part of the multidisciplinary team (GCP) Full-time dedicated podiatrist specialising in rheumatology is essential (GCP)	Access to podiatry should be available according to patient need (GCP) Podiatry services should provide specific and dedicated service for diagnosis, assessment and management of foot problems associated with RA (GCP) Timely intervention for acute problems is important (GCP)	Annual foot review and assessment is recommended for patients at risk of developing serious complications in order to detect problems early (GCP) Appropriate lower limb assessment for neurological and vascular status needed (GCP) Assessment of lower limb mechanics and foot pressures should occur (B)	Orthoses are an important and effective intervention in RA (B)	There should be a provision of specialist footwear if needed (B)
Clinical guidelines for the diagnosis and management of early rheumatoid arthritis [50]	Podiatry is part of the multidisciplinary team (GCP)	GPs should support access to appropriate foot care for patients with RA (GCP)	Annual foot review and assessment recommended for patients at risk of developing serious complications in order to detect problems early (GCP)	Appropriate foot orthoses are an important and effective intervention for RA (B)	
Management of early rheumatoid arthritis [51]				Custom made insoles can relieve pain (A)	Orthopaedic footwear that offers sufficient comfort, mobility and stability (A)
SIGN 123 Management of early rheumatoid arthritis [53]	Podiatry is part of the multidisciplinary team (GCP)	‘Good practice’ to offer all patients with early RA a podiatry referral (GCP)		Some evidence for the efficacy of foot orthoses for comfort, stride speed and stride length (C)	Appropriate footwear for comfort, mobility, and stability is well recognised in clinical practice but little available evidence (GCP)

A = Grade of recommendation based on systematic reviews; B = Grade of recommendation based on randomised controlled trials; C = Grade of recommendation based on quasi-experimental studies; D = Grade of recommendation based on non-experimental descriptive studies; GCP = Good Clinical Practice based on expert opinion

**Table 3:** Recommended guidelines for established RA

Guidelines	Multi-disciplinary team care
ARMA Standards of care for people with inflammatory arthritis [33]	People with inflammatory arthritis should have ongoing access to local multi-disciplinary team (GCP)
	Podiatry is part of the multi-disciplinary team (GCP)
Ottawa Panel evidence-based clinical practice guidelines for electrotherapy & thermotherapy interventions in the management of RA in adults [40]	
Structural evaluation in the management of patients with RA: Development of recommendations for clinical practice based on published evidence and expert opinion [44]	
BSR Guidelines on standards of care for persons with RA [46]	Podiatry is part of the multi-disciplinary team (GCP)
	Early referral for surgical opinion if required (GCP)
PRCA Standards of care for people with MSK foot health problems [34]	
Diagnosis and Treatment of RA [42]	
Clinical Practice Guidelines for non-drug treatment (excluding surgery) in RA [43]	
BSR and BHPR Guidelines for the management of RA (after the first 2 years) [48]	
NICE RA National clinical guideline for management and treatment in adults [49]	
Clinical practice guidelines for the management of RA in Spain [52]	
NWCEG Guidelines for the management of the foot health problems associated with RA [35]	Referral for surgery opinion should be offered as an alternative to therapeutic footwear referral (GCP)
	Optimum ulcer management can only be achieved by a holistic and integrated multi-disciplinary team approach (GCP)
	Contact the patient’s consultant/CNS immediately if the patient is being managed with biologic therapy and develops an ulcer and/or infection (GCP)
	Red flags requiring urgent referral–tendon rupture, septic arthritis, suspicion of cancer (GCP)
EULAR recommendations for the use of imaging of the joints in the clinical management of RA [41]	
Guidelines	Access to foot healthcare
ARMA Standards of care for people with inflammatory arthritis [33]	All people with a sudden ‘flare-up in their condition should have direct access to specialist advice and the option for early review with the appropriate multi-disciplinary team member (GCP)
Ottawa Panel evidence-based clinical practice guidelines for electrotherapy & thermotherapy interventions in the management of RA in adults [40]	
Structural evaluation in the management of patients with RA: Development of recommendations for clinical practice based on published evidence and expert opinion [44]	
BSR Guidelines on standards of care for persons with RA [46]	When clinically indicated access to podiatry should be available within 6 weeks of referral (GCP)
PRCA Standards of care for people with MSK foot health problems [34]	Timely access to foot healthcare – diagnosis, assessment and management (GCP)
	Adequate information/education should be given for self-management and signs/symptoms of deterioration in foot health and need to access specialist help promptly (GCP)
Diagnosis and Treatment of RA [42]	
Clinical Practice Guidelines for non-drug treatment (excluding surgery) in RA [43]	Every patient with RA should be informed of the rules of foot hygiene and of potential benefit of referral to a podiatrist (GCP)
	A podiatrist should be consulted to treat nail anomalies and hyperkeratoses on the feet of patients with RA (GCP)
BSR and BHPR Guidelines for the management of RA (after the first 2 years) [48]	
NICE RA National clinical guideline for management and treatment in adults [49]	All patients with RA and foot problems should have access to a podiatrist (GCP)
Clinical practice guidelines for the management of RA in Spain [52]	
NWCEG Guidelines for the management of the foot health problems associated with RA [35]	Referral to a podiatrist is an integral part of the early management of RA patients (GCP)
EULAR recommendations for the use of imaging of the joints in the clinical management of RA [41]	
Guidelines	Foot health assessment/review
ARMA Standards of care for people with inflammatory arthritis [33]	
Ottawa Panel evidence-based clinical practice guidelines for electrotherapy & thermotherapy interventions in the management of RA in adults [40]	
Structural evaluation in the management of patients with RA: Development of recommendations for clinical practice based on published evidence and expert opinion [44]	Investigations to monitor course of RA should include radiographs of forefeet and should be done every 6 months in the first year, then every year to the third year and every 2–4 years thereafter (GCP)
BSR Guidelines on standards of care for persons with RA [46]	
PRCA Standards of care for people with MSK foot health problems [34]	Foot healthcare providers must understand consequences of systemic disease on the feet and be able to identify warning signs that require timely referral to specialist medical care (GCP)
	Foot health assessment should occur within 3 months of diagnosis–doesn’t have to be done by foot health specialist (GCP)
	Annual review of foot health needs are desirable–doesn’t have to be done by foot health specialist (GCP)
	Where there is substantial change (better/worse) in disease activity, foot health should be reviewed (GCP)
Diagnosis and Treatment of RA [42]	
Clinical Practice Guidelines for non-drug treatment (excluding surgery) in RA [43]	Feet, footwear and orthoses should be regularly examined (GCP)
BSR and BHPR Guidelines for the management of RA (after the first 2 years) [48]	
NICE RA National clinical guideline for management and treatment in adults [49]	All patients with RA and foot problems should have access to a podiatrist for assessment and periodic review of their foot health needs (GCP)
Clinical practice guidelines for the management of RA in Spain [52]	
NWCEG Guidelines for the management of the foot health problems associated with RA [35]	All patients should be referred for foot health assessment with 3 months of diagnosis with RA (GCP)
	All people with RA and foot problems should have access to a podiatrist for assessment and periodic review of their foot health needs (GCP)
	Patients with RA diagnosis should be assessed as soon as possible after diagnosis for lower limb and foot structural problems (GCP)
EULAR recommendations for the use of imaging of the joints in the clinical management of RA [41]	Feet x-rays initial imaging technique to detect damage. Ultrasound and/or MRI should be considered if x-rays do not show damage and may be used to detect earlier damage (GCP)
	MRI or ultrasound detected synovitis and joint damage detected by x-rays, MRI or ultrasound can be considered for prediction of further joint damage (C)
	Periodic evaluation of joint damage should be considered. MRI (and possibly ultrasound) can be used to monitor disease progression (C)
	MRI and ultrasound detected inflammation predicts subsequent joint damage, even with clinical remission and can assess persistent inflammation (C)
Guidelines	Orthoses/insoles/splints
ARMA Standards of care for people with inflammatory arthritis [33]	
Ottawa Panel evidence-based clinical practice guidelines for electrotherapy & thermotherapy interventions in the management of RA in adults [40]	
Structural evaluation in the management of patients with RA: Development of recommendations for clinical practice based on published evidence and expert opinion [44]	
BSR Guidelines on standards of care for persons with RA [46]	
PRCA Standards of care for people with MSK foot health problems [34]	
Diagnosis and Treatment of RA [42]	Insoles may have a beneficial effect on pain in people with RA and foot complaints (A)
Clinical Practice Guidelines for non-drug treatment (excluding surgery) in RA [43]	Customised toe splints may be preventive, corrective or palliative to enable the wearing of shoes (GCP)
	Customised orthotic insoles are recommended in the case of weight-bearing pain or static foot problems (GCP)
	Orthoses should be regularly examined (GCP)
	Limited evidence for the use of foot orthoses - no consensus regarding choice of orthoses but reduction of pain and improved function of the foot are reported (A)
BSR and BHPR Guidelines for the management of RA (after the first 2 years) [48]	Functional insoles should be available to all people with RA if indicated (A)
NICE RA National clinical guideline for management and treatment in adults [49]	Insoles may have a beneficial effect on pain in people with RA and foot complaints (A)
Clinical practice guidelines for the management of RA in Spain [52]	Hard orthotics improve pain in the hindfoot in the initial phase of the disease (A)
	Use of a special model can prevent the development and progression of hallux valgus (A)
	All patients with RA and foot pain should be considered for foot orthoses advice, irrespective of disease duration (B)
NWCEG Guidelines for the management of the foot health problems associated with RA [35]	Patients with established foot deformity should be assessed for accommodative foot orthoses (C)
	Functional foot orthoses should be provided where tarsal joints are unaffected (B)
	Accommodative/cushioned orthoses should be provided when structural foot deformity, painful symptoms and activity restriction present (C)
EULAR recommendations for the use of imaging of the joints in the clinical management of RA [41]	
Guidelines	Therapeutic footwear
ARMA Standards of care for people with inflammatory arthritis [33]	
Ottawa Panel evidence-based clinical practice guidelines for electrotherapy & thermotherapy interventions in the management of RA in adults [40]	
Structural evaluation in the management of patients with RA: Development of recommendations for clinical practice based on published evidence and expert opinion [44]	
BSR Guidelines on standards of care for persons with RA [46]	
PRCA Standards of care for people with MSK foot health problems [34]	
Diagnosis and Treatment of RA [42]	Specially selected footwear may have a beneficial effect on pain in people with RA and foot complaints (B)
	Prescribing shoe adjustments and provisions must be considered in patients with RA and foot complaints (B)
Clinical Practice Guidelines for non-drug treatment (excluding surgery) in RA [43]	Patients should be advised about footwear (GCP)
	Footwear should be regularly examined (GCP)
	Extra-width off-the-shelf or therapeutic thermoformed shoes are recommended when the feet are deformed and painful, if shoes are difficult to put on, or other footwear types have failed (C)
	Such shoes reduce pain on walking and improve functional capacity (GCP)
	Palliative customised therapeutic shoes may be prescribed when feet are seriously affected (GCP)
BSR and BHPR Guidelines for the management of RA (after the first 2 years) [48]	Semi-rigid orthotic supportive shoes can be effective for metatarsalgia–reduction in pain, disability, and improvement in activity as measured by the FFI have been reported (B)
NICE RA National clinical guideline for management and treatment in adults [49]	Therapeutic footwear should be available to all people with RA if indicated (D)
Clinical practice guidelines for the management of RA in Spain [52]	Shoes with extra width improve the result of orthotics (A)
NWCEG Guidelines for the management of the foot health problems associated with RA [35]	All patients with RA and foot pain should be considered for therapeutic footwear advice, irrespective of disease duration (B)
	Patients with established foot deformity should be assessed for accommodative footwear advice/specialist footwear (B)
	Footwear assessment and advice should be given to all patients (GCP)
	Patients struggling with retail footwear due to deformity should be offered the option of referral for therapeutic footwear. They should be informed of potential benefits and limitations of this footwear in respect to cosmesis (B)
EULAR recommendations for the use of imaging of the joints in the clinical management of RA [41]	
Guidelines	Other treatments
ARMA Standards of care for people with inflammatory arthritis [33]	
Ottawa Panel evidence-based clinical practice guidelines for electrotherapy & thermotherapy interventions in the management of RA in adults [40]	Low-level laser therapy is beneficial for pain relief in the feet (B)
Structural evaluation in the management of patients with RA: Development of recommendations for clinical practice based on published evidence and expert opinion [44]	
BSR Guidelines on standards of care for persons with RA [46]	
PRCA Standards of care for people with MSK foot health problems [34]	
Diagnosis and Treatment of RA [42]	
Clinical Practice Guidelines for non-drug treatment (excluding surgery) in RA [43]	
BSR and BHPR Guidelines for the management of RA (after the first 2 years) [48]	
NICE RA National clinical guideline for management and treatment in adults [49]	
Clinical practice guidelines for the management of RA in Spain [52]	
NWCEG Guidelines for the management of the foot health problems associated with RA [35]	Callus should be assessed in relations to symptoms and causative factors before debridement is considered (GCP)
	Fungal infections (of the nail and skin) must be investigated and treated. If left untreated they can lead to ulceration and secondary bacterial infection. Discussion with the patient’s GP or consultant advised before systemic treatment is instigated (GCP)
	Consultant advice should be taken on ingrown toenails if the patient is being managed with a biological therapy and where there are signs of clinical infections and/or need for nail surgery (GCP)
	Patient education should include foot health self management advice and if necessary demonstration, explanation of foot problems and their impact on the individual, information on general disease management and signposting for future foot health needs (GCP)
	Consider steroid injection therapy for targeting localised, inflamed joints when the general disease is controlled (only in absence of sepsis) (GCP)
	Injection therapy should be seen as an adjunct to conventional podiatric management in combination with attempts to correct any structural deformity using orthoses (GCP)
EULAR recommendations for the use of imaging of the joints in the clinical management of RA [41]	

A = Grade of recommendation based on systematic reviews; B = Grade of recommendation based on randomised controlled trials; C = Grade of recommendation based on quasi-experimental studies; D = Grade of recommendation based on non-experimental descriptive studies; GCP = Good Clinical Practice based on expert opinion

Low quality guidelines not recommended for use included a number of foot care recommendations that concurred with high quality guidelines or low quality guidelines that were recommended for use. For early RA, the recommendations were use of orthoses/insoles/splints for pain relief [20]. For established RA, the recommendations were podiatry is part of the multidisciplinary team [21, 22], joint protection with orthoses/insoles/splints [23–26], and radiographs of the feet on initial diagnosis and then annually to monitor disease progression [24]. No foot and ankle care recommendations were found in the guidelines not recommended for use that were not also present in guidelines recommended for use.

## Discussion

Foot and ankle care features in many RA management guidelines, that following appraisal would be recommended for clinical use. Five guidelines were fully recommended, twelve were recommended with modifications, and seven were not recommended. There were four and six high quality guidelines for the management of early RA and established RA respectively. There were also one and six recommended low quality guidelines for early and established RA respectively. Recommendation domains were multidisciplinary team care, access to foot healthcare, foot health assessment/review, orthoses/splints/insoles, therapeutic footwear, and other foot care treatments. The strength of the grade of recommendation (based on levels of evidence) for each domain was predominantly ‘good clinical practice’. This was with the exception of foot orthoses and therapeutic footwear, which had higher grades of recommendation underpinned by a limited number of systematic reviews and randomised controlled trials.

Many of the guidelines advocated podiatry as part of the multidisciplinary team and included specific foot and ankle management options. This is due to: 1) persistent foot problems can still occur even after reaching clinical remission [9–11]; 2) people with increased disease states may have mechanical foot impairments needing treatment in conjunction with systemic management; and 3) people who do not respond to or are ineligible for biological therapy may continue to have active foot involvement [27]. Unfortunately, these guidelines offer no guidance as to how foot and ankle care should be incorporated into the multidisciplinary team.

As far as we are aware, this is the only guideline appraisal completed for foot and ankle care as part of RA management. There is minimal previous work completed within rheumatology, where only one systematic appraisal was identified for lower limb osteoarthritis [28] and one for knee and hip management in osteoarthritis [29]. No appraisals of guidelines were found for other areas of foot and ankle care or podiatry.

Clinical practice guidelines need to be appraised for quality as they are used to inform appropriate healthcare and assist in clinical decision making [1]. However, this is only possible if the guidance is good quality [2, 4]. Guidelines that do not meet an appropriate standard might actually be detrimental to patient health as they might recommend care that could be harmful. This was not the case in this appraisal, as non-recommended guidance concurred with recommended guidelines meaning these particular recommendations could be used clinically. However, ideally the recommended guidelines should be utilised in preference. Additionally, it should be noted that these are only guidelines, and health practitioners must be careful not to place too much emphasis on them [1]. They take time to develop and newer evidence may become available which is not included in the guidelines. This consideration is particularly important in this case, as there was variability in the evidence included in each individual guideline as a consequence of differing development strategies and year of publication. Additionally, clinical experience is still important, especially in areas where limited evidence is present [2]. This is also seen in the guidelines where many recommendations are based on ‘good clinical practice’ or ‘expert opinion’.

The availability of multiple guidelines presents opportunities for varied use and implementation across clinical practice, and consequently this may impact on the delivery of care. There is evidence to support this in podiatry, where guidelines are better known and understood by those in specialist roles in comparison to non-specialists [30]. This clinical appraisal presents recommendations based on quality indicators so may be useful to standardise the delivery of evidence based care.

High quality guidelines tended to be from government agencies or established guideline groups. The higher assessment was most likely due to the more systematic approach taken to develop the guidelines. This was consistent with a study that looked at the characteristics of high quality guidelines by evaluating 86 clinical practice guidelines from eleven different countries [31]. Conversely, the low quality guidelines were developed by smaller specialist groups. This was also consistent with another study that found guidelines developed by specialist groups were unsatisfactory when critically appraised [32].

Applicability of the guidelines was a low scoring domain and many of the guidelines that needed modifications required them in this area. This differed to a study that found that applicability was decreased in the high quality guidelines as there was more emphasis on development methodology rather than the effectiveness of the guidelines in clinical practice [31]. However, it is difficult to determine if absence of applicability from the guidelines was due to applicability not being considered or referral to it was just simply omitted. Applicability is important as it includes the feasibility of implementing the guidelines within the healthcare sector. There is limited benefit in a guideline advocating particular foot and ankle health recommendations if it is not feasible for them to be implemented. This could be from financial, staffing or time perspectives and/or restrictions. This is particularly an issue currently within the UK National Health Service due to funding restrictions. On a positive note, many of the specialist groups did provide good clinical practice points which can help to standardise delivery of care [33–35].

The stakeholder domain generally scored quite highly. However, it was noticed during appraisal that patient involvement was not always present, even though they are major stakeholders in clinical care and should be actively involved [36]. Additionally, many of the development groups for the recommended guidelines did not include foot care specialists. This was even though it has previously been highlighted that including practitioners in the feasibility and acceptability of guidelines improves the overall effectiveness of the guideline [37].

Few of the recommended guidelines were specifically related to early RA. This is even though there has been a paradigm shift for a more targeted and aggressive approach that takes advantage of the ‘window of opportunity’ for all management strategies including systemic and non-pharmacological interventions [27, 38]. However, the limited number of early RA guidelines including foot and ankle care may reflect an overall lack of evidence underpinning proposed paradigm shifts in foot care [39].

It should be noted that two of the guidelines were classified as standards and did state that they were not clinical practice guidelines [33, 34]. However, the recommendations in these standards are often implemented clinically. Additionally, the statement regarding being a standard and not a guideline was very hard to find within the documents.

Whilst foot and ankle care recommendations were taken from the guidelines, the guideline in its entirety was appraised. The way to determine the percentage scores for each domain was specified by the AGREE II instrument. However, the overall score was determined by the reviewers and was applied in the same numerical way as each domain score. Overall, in determining quality of the guidelines, the domain requiring modification dictated the quality level. The rigour of development domain was deemed more important than other domains, because if modifications were needed for this domain then the whole guideline would need to be redone compared to small additions being made as modifications for the other domains. This was why some guidelines were high quality with modifications compared to low quality with modifications, even though the overall assessment score was the same. This was also the case for low quality guidelines recommended for use with modifications compared to low quality guidelines not recommended for use.

The limitations of this critical appraisal mainly related to the appraisal instrument used. The AGREE II instrument has not been tested for reliability. However, the predecessor is a valid and reliable instrument, and the AGREE II instrument has good construct validity and the changes made to create it were completed to increase the instrument measurement properties [18]. The use of percentages meant that guidelines may have had the same overall score. However, this could mean that a guideline may have had similar scores for each domain or alternatively, some domains had higher and/or lower scores than others. Additionally, no guidance was given with the AGREE II instrument about how to determine high and low quality. This was decided collectively by the reviewers to reduce bias in the assessment.

Only new editions of guidelines were appraised, which meant that comparisons between different editions of guidelines was not possible. However, this was not deemed necessary as newer editions superseded previous editions. Additionally, another study showed there is a small amount of improvement over time between editions [31]. As there was no language restriction for the guidelines, there may have been translation issues for those not in English. However, one of the reviewers was multilingual which helped to reduce issues with translation.

## Conclusion

Many recommendations for foot and ankle care were present in the clinical practice guidelines that were appraised and recommended for use. This appraisal has identified a wide breadth of recommendations within these guidelines, from multidisciplinary team care and service access to assessment and specific interventions. Unfortunately, supporting evidence in the guidelines was low quality overall, with grades of recommendations predominantly being ‘good clinical practice’ or ‘expert opinion’. Whilst the recommendations identified show the current minimum clinical standard, more research investigating foot and ankle management in RA is needed prior to inclusion in clinical practice guidelines.

## Abbreviations

AGREE: Appraisal of guidelines for research and evaluation
MeSH: Medical subject heading
RA: Rheumatoid arthritis
